# Fractionating auditory priors: A neural dissociation between active and passive experience of musical sounds

**DOI:** 10.1371/journal.pone.0216499

**Published:** 2019-05-03

**Authors:** Marina Kliuchko, Elvira Brattico, Benjamin P. Gold, Mari Tervaniemi, Brigitte Bogert, Petri Toiviainen, Peter Vuust

**Affiliations:** 1 Center for Music in the Brain (MIB), Department of Clinical Medicine, Aarhus University & The Royal Academy of Music Aarhus/Aalborg (RAMA), Aarhus, Denmark; 2 BioMag Laboratory, HUS Medical Imaging Center, University of Helsinki and Helsinki University Hospital, Helsinki, Finland; 3 Montreal Neurological Institute, McGill University, Montreal, Canada; 4 Cicero Learning, Faculty of Educational Sciences, University of Helsinki, Helsinki, Finland; 5 Cognitive Brain Research Unit, Department of Psychology and Logopedics, Faculty of Medicine, University of Helsinki, Helsinki, Finland; 6 Department of Music, Art and Culture Studies, University of Jyväskylä, Jyväskylä, Finland; Universidad de Salamanca, SPAIN

## Abstract

Learning, attention and action play a crucial role in determining how stimulus predictions are formed, stored, and updated. Years-long experience with the specific repertoires of sounds of one or more musical styles is what characterizes professional musicians. Here we contrasted active experience with sounds, namely long-lasting motor practice, theoretical study and engaged listening to the acoustic features characterizing a musical style of choice in professional musicians with mainly passive experience of sounds in laypersons. We hypothesized that long-term active experience of sounds would influence the neural predictions of the stylistic features in professional musicians in a distinct way from the mainly passive experience of sounds in laypersons. Participants with different musical backgrounds were recruited: professional jazz and classical musicians, amateur musicians and non-musicians. They were presented with a musical multi-feature paradigm eliciting mismatch negativity (MMN), a prediction error signal to changes in six sound features for only 12 minutes of electroencephalography (EEG) and magnetoencephalography (MEG) recordings. We observed a generally larger MMN amplitudes–indicative of stronger automatic neural signals to violated priors–in jazz musicians (but not in classical musicians) as compared to non-musicians and amateurs. The specific MMN enhancements were found for spectral features (timbre, pitch, slide) and sound intensity. In participants who were not musicians, the higher preference for jazz music was associated with reduced MMN to pitch slide (a feature common in jazz music style). Our results suggest that long-lasting, active experience of a musical style is associated with accurate neural priors for the sound features of the preferred style, in contrast to passive listening.

## Introduction

Our ability to learn relies on sustained, active engagement with the sensory stimulation utilized for predicting future events and reducing errors. According to the predictive coding theory [1,2], the brain functions as a probabilistic machine learning to predict the sensory environment and updating predictions based on any new experience of incoming stimuli. In auditory perception, experience modifies top-down predictions for sounds so that these predictions eventually become more precise in order to minimize uncertainty. Hence, when a sound stimulus follows a predictable pattern, there is no need to recruit more neural resources whereas when a novel stimulus mismatches a pattern learned from previous exposure, additional neuronal assemblies produce an error signal to update the prior.

The validity of the predictive coding theories in explaining cortical functions in the auditory domain has been tested using neurophysiology. Findings support the view of hierarchical feedback and feedforward processes allowing auditory learning. Specifically, attenuated N1 cortical responses to repeated sounds have been linked to the online updating of neural predictions [3], whereas the change-related neural response called mismatch negativity (MMN) have been proposed to index an error signal in the predictive coding of the auditory environment (a mismatch between incoming sensory information and prediction) automatically elicited in the auditory cortex [1,4–8]. This MMN error signal indexes the process of updating prior predictions according to sensory feedback, hence leading to auditory learning [9].

Prediction errors depend on both the content and precision of a prediction, which can be formed in the short- or long-term period [10,11]. Moreover, forming priors may occur by means of passive exposure or as a result of a concentrated and attentive state during learning or while using new information and skills. An illustrative example of priors resulting from active as well as passive exposure to novel information can be drawn from music. Experience with musical sounds results in implicitly learning the acoustic features and conventions of the musical system and style to which one is most exposed even if no musical training takes place [12]. Happening over the course of life, this results in better schematic memory, e.g., for specific chord successions [13,14] and rhythm patterns [15], as well as higher affective judgments for the music of one’s own culture (e.g., Western classical music or Indian raga) [16]. This implicit learning of one’s own musical culture is already present in early childhood [17,18] and can be imprinted even before birth [19].

At the other end of this musical experience spectrum are musicians who focus their selective attention on music daily, study it intensively both in theory and practice, engage with it emotionally, and do this for thousands of hours [20]. This intense and attentive experience with music is accompanied by behavioural, neurophysiological and anatomical changes in the musicians’ brain (for reviews, see [21–24]). Compared to non-musicians, musically trained individuals have an increased volume of auditory, motor and visual-spatial cortical areas [25–28], cerebellum [29] and corpus callosum [30]. Some studies have linked changes in regional brain anatomy with musical proficiency and functional differences in processing of pure tones [27] and spectrotemporal processing of music [28] and speech [31] sounds. Training-induced changes in the brain can be traced already after fifteen months of instrumental practice in children [32].

The distinct characteristics of different instruments and styles play a putative role in music-derived neuroplasticity. Listening to a melody played by an instrument of one’s own expertise engages auditory-perceptual, motor and self-relevance brain networks in musicians to a larger extent than when listening to another instrument playing the same melody [33,34]. Moreover, the spectral differences in the sound of an instrument played are reflected in the structural organization of the auditory cortex and Heschl’s gyrus in particular [35] as well as the timbre of one’s own instrument elicits a stronger cortical representation compared to a synthetic or different instrument sound [36]. Familiarity with a particular musical style, too, influences perceptual skills of musicians, posing specific demands to their musical practice: as distinct from classical and band musicians, jazz players have enhanced neural discrimination of pitch slide, which is a typical attribute of jazz music and atypical to non-improvisational musical genres [37]. A study by Tervaniemi and colleagues [38] showed selective discrimination profiles in auditory-cortex MMN responses of classical musicians to mistuning and timing of melodies, while MMN of jazz musicians were stronger to timing, melody contour and transposition. Based on these findings one can speculate that practice style and the interactive performance tradition of jazz music demands stronger priors about the melodic changes as opposed to playing closely following the score in the classical music tradition. However, it remains to be determined how the specific content of priors derived from distinct learning processes might alter sensory experience.

In this study, we asked whether active experience of classical and jazz music (as derived from instrumental training in music schools and intense, long-term practicing and performing) would be more powerful in refining the prediction error signal for feature changes in tone patterns as opposed to the passive preference for the style (listening to music with minimal or no active practice, theoretical knowledge and formal musical training). To our aims, we measured the MMN non-invasively from the surface of the scalp by means of electroencephalography (EEG) and magnetoencephalography (MEG). We used a modified version [39,40] of fast, musical multi-feature MMN paradigm first introduced by Vuust and colleagues [41]. The paradigm was shown to be efficient in demonstrating differences in neural discriminatory skills between professional musicians representing different musical styles, namely rock/pop, jazz and classical musicians [37]. We expected to obtain enhanced MMN amplitudes (indexing more accurate prediction errors) for the features specific to the style played by professional musicians and we also expected to see a differential effect of the type of listening experience (active or passive) on the MMN responses.

## Materials and methods

### Participants and musical backgrounds

The experimental procedure for this study was included in the research protocol “Tunteet” (Emotions) investigating different aspects of auditory processing with several experimental paradigms. The findings related to different hypothesis are reported in separate papers (e.g. [42–45]). Ethical permission was granted by the Ethics Committee of the Hospital District of Helsinki and Uusimaa (approval number: 315/13/03/00/11, obtained on March the 11th, 2012). All procedures were conducted in agreement with the ethical principles of Declaration of Helsinki. Participants signed an informed consent on arrival to the lab and received a compensation for their time after the experimental session.

140 volunteers with reported normal hearing and no history of neurological disease participated in ‘Tunteet’ data collection; 120 of them (54 men, 66 women) were presented with a research paradigm in question and comprised a subset analyzed for the current paper. Due to technical problems during data acquisition, ten recordings lucked EEG data. Eleven MEG and 11 EEG recordings were left out of the analysis because of less than 100 trials per stimulus accepted for the analysis in the preprocessing stage (10% of the current data subset).

Complying with the recommendations for studying music-derived neuroplasticity [46], we assessed factors that might affect neural responses to sounds by surveying the demographics and musical backgrounds of our participants. An initial screening was obtained with a musical background questionnaire [47] handed prior to the measurement. In addition, we asked subjects to fill in an online form called Helsinki Inventory of Music and Affective Behaviors (HIMAB; [48,49]). Among other scales, HIMAB included musical background assessment, the Listening to Music questionnaire [50] measures of weekly time spent on active and passive listening to music, a question on genre self-determination for individuals playing music, the Short Test of Music Preferences (STOMP;[51]), and the On-line Test for identification of congenital amusia [52]. Subjects could complete it at home and they were given a research assistant’s phone number to contact should they have any questions while filling out the form. The completion of HIMAB was left to participants' choice depending on how much time they were willing to dedicate to the Tunteet protocol. Out of the current sample, 60 subjects completed HIMAB. Group performance on all musicality tests and its relation to MMN is reported in S1 Table.

Based on the information collected with the paper and on-line questionnaires, we grouped subjects according to their musical background, musical self-identification, and practiced musical styles: non-musicians (NM), amateur musicians (AM), jazz musicians (JM) and classical musicians (CM). The details on subjects’ musical background are described in Table 1. NM were subjects with fewer than three years of musical training or occasional practice not exceeding one hour a week. AM were self-taught or had some musical training on the level below professional education, and/or practiced music on a regular basis. Two of them were group outliers for the amount of music playing experience with 20 and 30 years of continuous engagement with music practice, and thus were excluded from the analysis. JM and CM had a degree in music, and were currently teaching and/or performing jazz or classical music, respectively. Both groups of professional musicians varied on the type of instrument they play. All of them, except for two CM, reported practicing more than one instrument or singing. JM and CM were comparable in length of training and playing an instrument, hours of weekly musical practice and amounts of active and passive listening to music. Nevertheless, these two groups were not balanced in gender: men were prevalent in JM while there were more women in CM group (Table 2).

**Table 1:** Musical background.

	Group			
Mean ± SD	NM	AM	JM	CM
**Years of training**	0.36 ± 0.70	4.79 ± 2.22	16.69 ± 8.77	17.67 ± 4.73
**Years of playing**	0.56 ± 0.97	7.00 ± 3.57	20.77 ± 6.00	22.73 ± 7.16
**Music onset (age)**	26.56 ± 17.02	13.28 ± 6.45	7.27 ± 3.04	7.40 ± 5.59
**Playing (h/week)**	0.04 ± 0.18	1.05 ± 1.85	16.23 ± 10.28	15.00 ± 12.79
**Active listening (h/week)**	5.54 ± 6.11	6.75 ± 6.54	8.75 ± 2.44	11.45 ± 11.51
**Passive listening (h/week)**	11.38 ± 7.35	14.25 ± 15.24	16.38 ± 13.70	15.10 ± 9.33
A type of an instrument played*				
** String**	4 (0)	6 (2)	4(2)	1(4)
** Keyboard**	4 (0)	14 (5)	4(4)	5(5)
** Bowed string**	0 (0)	2 (0)	1(1)	8(2)
** Voice**	5 (1)	1 (11)	1(5)	0(9)
** Wind/Brass**	1 (0)	2 (0)	2(5)	0(3)
** Percussion**	1 (0)	3 (1)	1(4)	1(2)

*Number of subjects playing a type of an instrument as their main (or secondary) instrument

**Table 2:** Demographic data of subjects.

Group	EEG				MEG			
	N	Gender	Age *	Handedness	N	Gender	Age*	Handedness
**NM**	40	20M/20F	27.9 ± 8.4	37R/3L/0A	47	21M/26F	28.3 ± 8.6	43R/4L/0A
**AM**	25	8M/17F	28.2 ± 9.3	24R/1L/0A	28	9M/19F	28.2 ± 8.8	27R/1L/0A
**JM**	13	11M/2F	30.1 ± 7.7	11R/1L/1A	13	11M/2F	30.1 ± 7.7	11R/1L/1A
**CM**	15	3M/12F	28.6 ± 8.0	14R/1L/0A	15	3M/12F	28.6 ± 8.0	14R/1L/0A

M–male, F–female; R–right-handed, L–left-handed, A–ambidexterity

*Mean ± SD.

Five of the recruited subjects were musicians actively playing and performing rock and/or pop music. As the number of these subjects was small, we could not include this group into the analysis and the data were omitted. The final data set included 102 MEG and 93 EEG recordings. Demographic details are presented separately for MEG and EEG samples in Table 2.

### Stimuli and procedure

We used a fast musical multi-feature MMN paradigm that consists of four-tone patterns arranged in an ‘Alberti bass’ music accompaniment figure typically found in Western tonal music. In the originally introduced version of the paradigm [37,41], every other pattern included one of the six types of deviant features, while the other patterns were ‘standard’. In the current study, we used a version of the musical multi-feature MMN paradigm [39,40] where the ‘standard’ patterns were omitted to create a more complex sounding sequence with rapid presentation of deviants as compared with the original paradigm by Vuust et al. [41]. We assumed that the higher demand of no-standard MMN paradigm may reveal finer details on how regular practice of a particular style and its associated stylistic features tune neural auditory discrimination.

**Fig 1:**
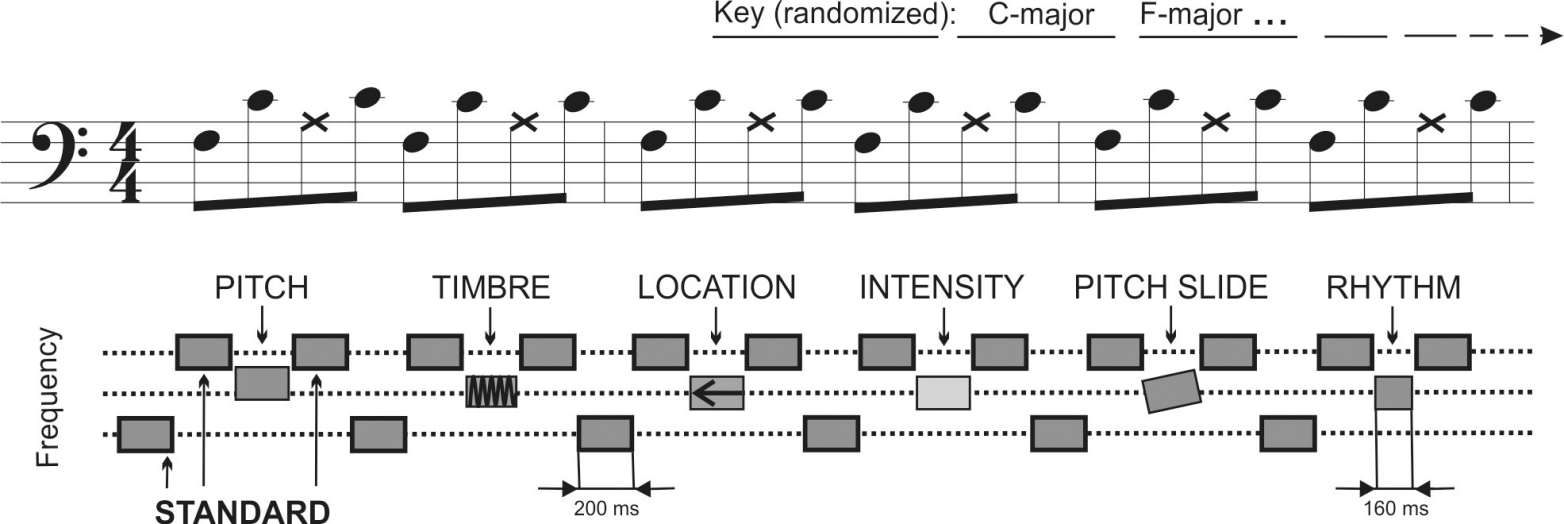
Stimuli. “Alberti Bass” played with piano tones in notation (top row) and schematic (bottom row) depiction. Each tone was 200 ms with an ISI of 5 ms. Each pattern of four notes includes deviant sound at the 3^rd^ position (X notes on the figure). Deviant order is randomized. The patterns are transposed to a different key every six bars, going through all major and minor keys during the presentation.

The no-standard musical multi-feature MMN paradigm is shown in Fig 1. Sound stimuli were generated using the sample sounds of Wizoo acoustic piano from the software sampler “Halion” in Cubase (Steinberg Media Technologies GmbH). The patterns were played in each of the 24 possible keys, changing every six patterns in pseudo-random order. The 3^rd^ tone of each pattern was a deviant of one of the six types: pitch, timbre, location, intensity, slide, or rhythm.

The deviants were created by modification of the sound in Adobe Audition (Adobe Systems Incorporated). The pitch deviant was a mistuning of a tone by 24 cents, tuned downwards in the major mode and upwards in the minor mode. The timbre used the ‘old-time radio’ effect provided with Adobe Audition using a 4 channel parametric equalizer (low shelf cutoff by −4.6 dB at 21.7 Hz; high shelf cutoff by −6.8 dB at 3354.9 Hz; 18.8 reduction at 83.7 Hz; 11 dB increase at 192.5 Hz; 11.6 dB increase at 623.1 Hz; 17.7 dB increase at 1663.7 Hz with a constant width of 1/4 of each of the frequencies; 3 dB overall amplitude reduction). The location deviant was generated by decreasing the amplitude of the right channel up to 10 dB, perceptually resulting in a sound coming slightly from a location centered to the left (∼70°) from the midline. The intensity deviant was made by reducing the original intensity by 6 dB. The slide deviant was made by sliding up the pitch to that of the standard from two semitones below. The rhythm deviant was created by shortening the third note by 40 ms. Thus, the following tone appeared earlier producing a change in a rhythmic contour. Sounds were amplitude normalized. Each tone was in stereo with 44.100 Hz sampling frequency. The duration of a single tone was 200 ms (except for the rhythm deviant lasting 160 ms) with ISI of 5 ms. Each deviant type was presented 144 times in pseudo-random order. The duration of the paradigm was 12 minutes. The sound examples can be found in [40].

The stimuli were presented with Presentation software (Neurobehavioral Systems, Albany, USA). Participants were comfortably seated in a chair with their head placed inside the helmet-like space of the MEG machine. The sound was delivered by a pair of pneumatic headphones. The loudness of the stimuli was kept at a comfortable level [40], which was personally adjusted for each subject prior to the MEG measurement. During the recording, subjects were watching a silenced movie of their own choice with subtitles. In the same experimental session, the subjects were presented with other experimental paradigms comprising Tunteet EEG/MEG protocol, which are and will be reported in separate papers [39,40,43,44].

### MEG/EEG data acquisition and analysis

The data were recorded with a 306-channel Vectorview whole head MEG device (Elekta Neuromag, Elekta Oy, Helsinki, Finland) and a compatible EEG system at the Biomag Laboratory of the Helsinki University Central Hospital. The MEG device had 102 pairs of planar gradiometers and 102 magnetometers built into a helmet-like device. For EEG recording, we used a 64-channel electrode cap connected to an amplifier for simultaneous EEG and MEG recordings. Electrooculography (EOG) electrodes were attached at the temples close to the external eye corners, above the left eyebrow and on the cheek below the left eye to monitor eye movements and blinks. The reference electrode was attached to the tip of the nose and the ground electrode was to the right cheek. F head position indicator coils were placed on top of the EEG cap. The location of the coils was determined respective to nasion and preauricular anatomical landmarks by Isotrack 3d digitizer (Polhemus, Colchester, VT, USA) to monitor a position of the head inside the MEG helmet. MEG/EEG data were recorded with a sample rate of 600 Hz. The recordings were done in an electrically and magnetically shielded room (ETS-Lindgren Euroshield, Eura, Finland).

The data were analyzed with BESA 6.0 software (BESA GmbH, Germany). First, EEG data were visually inspected and the maximum of six channels with noisy signals were interpolated. The data were further processed by an automatic eye-blink correction. Thereafter, the EEG and MEG responses were divided into epochs time-locked to the stimulus onset and baseline corrected. The epochs were 500 ms long including 100 ms of baseline prior to the stimulus onset. An epoch was automatically removed if it included an amplitude change exceeding the threshold of ±100 μV for EEG data, 1200 fT/cm for gradiometers and 2000 fT for magnetometers. The data file was excluded from further analysis when less than 100 trials of any type of a deviant were accepted. In the next step, the data were averaged according to the stimulus type. For the rhythm deviant, the 4^th^ note of a pattern was used for averaging, as this note made an interruption in the rhythmic structure, whereas the shorter 3^rd^ tone of the rhythm deviant pattern was excluded from the analysis. For all other deviants, the 3^rd^ tones of a pattern was used for averaging. The 1^st^, 2^nd^ and 4^th^ tones of the pattern (but only 1^st^ and 2^nd^ tones of the pattern with the rhythm change) were used for averaging as standard stimuli. The averaged waveform for the standard stimulus was subtracted from each deviant waveform. The resulting difference waveforms were analyzed in order to define the MMN and MMNm peaks.

MMN peak latency was automatically searched at Fz electrode separately for each type of a deviant in the time windows visually identified from the grand-averaged waveforms (100–250 ms for the timbre, the intensity and the rhythm deviants; 150–250 ms for the pitch deviant; 100–220 ms for the slide deviant; 70–150 ms for the location deviant). Since mastoid electrodes were not provided in the EEG system, we used inferior temporal electrodes TP9 and TP10 to evaluate the polarity reversal of MMN signal. The mean MMN amplitude (± 20 ms centered at the MMN peak) was automatically extracted on pairs of frontal and central electrodes (F3, F4, C3, C4) and inferior temporal electrodes acting as mastoids (TP9, TP10). We tested the significance of the MMN responses against the zero baseline for each deviant at Fz electrode and inferior temporal electrodes (TP9, TP10).

For MEG data, vector sums of gradiometer pairs were computed by squaring the MEG signals and calculating the square root of their sum. Then the individual areal mean curves for each subject and deviant type were obtained by averaging these vector sums over 16 symmetrical gradiometer pairs in the left and right temporal areas showing the maximal response. MMNm amplitudes were measured from the individual difference waveforms for each deviant and hemisphere by centering a 40-ms time window around the latency of the largest positive peak searched within the time windows identical to those used for EEG data.

To study the differences in the MMN/MMNm amplitudes between groups, deviant types, and distribution, we used mixed model ANOVA. For EEG data, the main analysis was done using Group (NM, AM, JM, CM) as the between-subjects factor and Feature (pitch, timbre, location, intensity, slide, rhythm), Laterality (F3, C3 and F4, C4) and Frontality (F3, F4 and C3, C4) as within-subjects factors. For MEG data, we performed ANOVA with Group (NM, AM, JM, CM) as between-subjects factor and Feature (pitch, timbre, location, intensity, slide, rhythm) and Laterality (left, right) as within-subjects factors. Analogous ANOVAs were run for each deviant separately to follow up Feature x Group interactions. All ANOVAs are reported with Greenhouse-Geisser corrected *P* values and the original degrees of freedom. The effect sizes are presented as partial eta-squared, *η*_*p*_^*2*^. Paired post-hoc comparisons were conducted using Bonferroni correction and only corrected P values are reported.

We used the scores of preference for jazz and classical music to test the effects of mere preference for a musical style on the neuronal sound feature discrimination in NM and AM. Preference for musical style was calculated as the sum of two ratings given by subjects on a seven-point Likert scale to (1) how much they liked, and (2) how familiar they were with each of the given musical styles. We correlated the preference scores for jazz and classical music with MMNm amplitudes. We employed a correlation analysis rather than a cross-sectional design because participants more often preferred several musical styles rather than a single one, which did not allow for designing groups with a clear preference for either jazz or classical music. Moreover, since preference for these two musical styles of interest were correlated (r = 0.336, p = 0.004), we opted to use Pearson’s partial correlation analysis. The contribution of musical experience was evaluated by correlating MMNm amplitude with years of playing music, which describes the musical experience of NM and AM who might or might not have formal musical training to the fullest.

## Results

### EEG data

**Fig 2:**
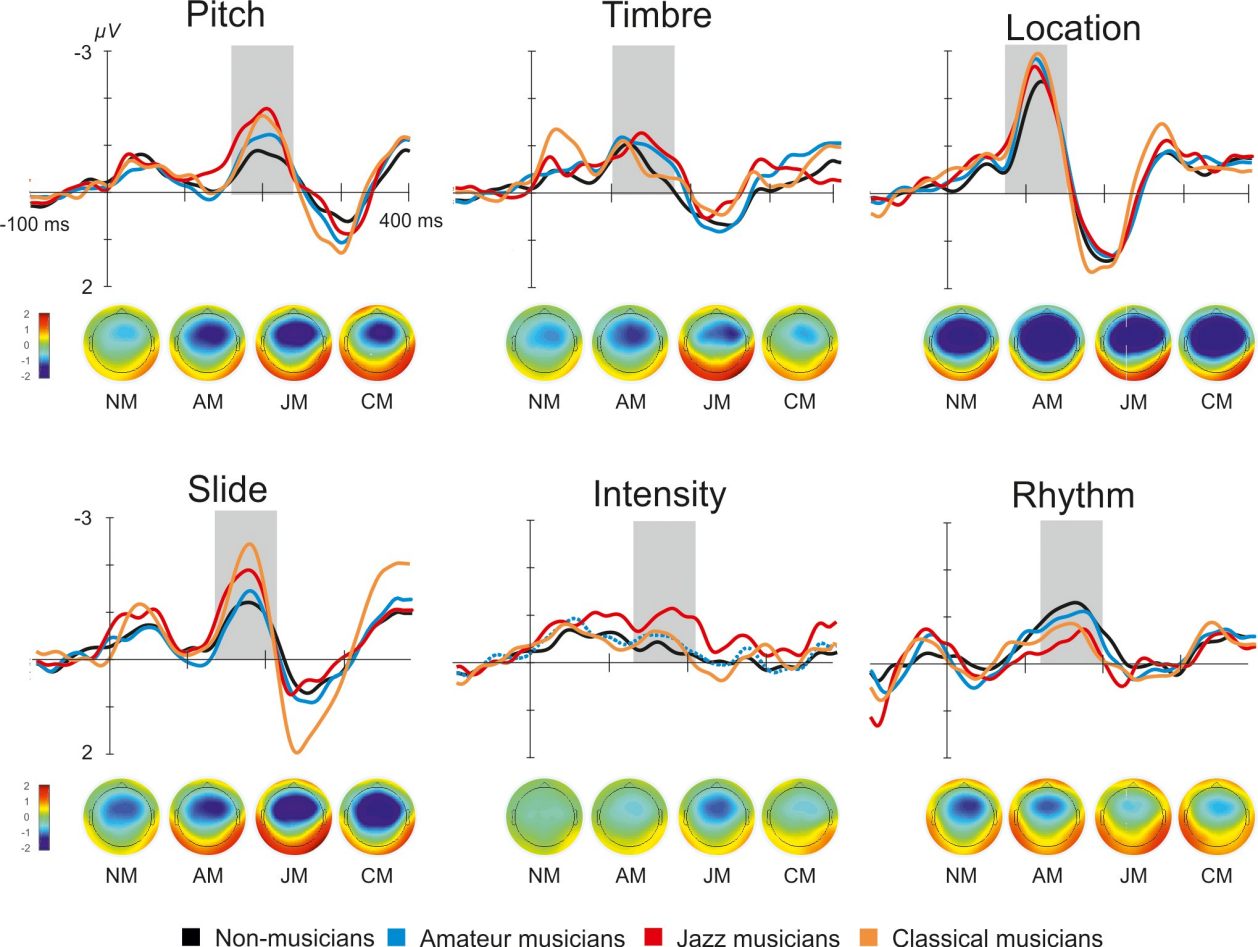
Grand averaged difference waveforms and topographic maps of MMN for four groups and six deviants recorded at Fz electrode. Grey area marks MMN peak.

MMNs to all six types of deviants are illustrated in Fig 2. Significant MMN responses were elicited by all deviants (for the measured *p* values see Table 3). Positive MMN reversal was registered for all deviants at both inferior temporal electrodes (p < 0.05).

**Table 3:** MMN amplitudes and latencies to different sound features recorded at Fz electrode and mastoids.

	Amplitude	*SD*	*t*	*dF*	*p*	Latency	*SD*
**Pitch**							
** **Fz	-1.37	1.07	-12.36	92	**< 0.001**	200	2.3
** **TP9	0.94	1.01	8.78	92	**< 0.001**		
** **TP10	1.33	0.98	12.61	92	**< 0.001**		
**Timbre**							
** **Fz	-1.25	1.11	-10.98	92	**< 0.001**	127	2.7
** **TP9	0.67	0.83	7.72	92	**< 0.001**		
** **TP10	1.16	0.82	13.11	92	**< 0.001**		
**Location**							
** **Fz	-2.57	1.47	-16.88	92	**< 0.001**	118	1.8
** **TP9	0.97	0.90	10.69	92	**< 0.001**		
** **TP10	1.46	1.05	13.73	92	**< 0.001**		
**Intensity**							
** **Fz	-1.02	1.00	-9.85	92	**< 0.001**	172	3.1
** **TP9	0.51	0.77	6.42	92	**< 0.001**		
** **TP10	0.92	0.71	12.38	9	**< 0.001**		
**Slide**							
** **Fz	-1.72	1.20	-13.89	92	**< 0.001**	178	2.3
** **TP9	1.20	1.33	8.65	92	**< 0.001**		
** **TP10	1.53	1.17	12.37	92	**< 0.001**		
**Rhythm**							
** **Fz	-1.38	0.94	-14.29	92	**< 0.001**	163	2.7
** **TP9	1.28	0.81	14.74	92	**< 0.001**		
** **TP10	1.48	0.89	15.48	92	**< 0.001**		

Significant *p-*values are shown in bold.

MMN latency varied significantly according to Feature (*F*_5, 445_ = 165, *p* < 0.0001, *η*_*p*_^*2*^ = 0.65). The shortest latency was found to the location deviant (*p* < 0.0001) and the MMN with the longest latency was elicited by pitch deviant (*p* < 0.0001) (Table 3). Group did not have any effect on MMN latency (*F* < 1).

The results of ANOVA analysis with MMN amplitudes showed no main effect of Group (p = 0.291), but a significant interaction of Group x Feature (*F*_15, 445_ = 2.56, *p* = 0.02, *η*_*p*_^*2*^ = 0.081). Separate follow-up ANOVAs for each deviant suggested Group effects on MMN amplitudes to slide (*F*_3, 89_ = 4.34, *p* = 0.007, *η*_*p*_^*2*^ = 0.128) and rhythm (*F*_3, 89_ = 2.97, *p* = 0.036, *η*_*p*_^*2*^ = 0.091). However, the Bonferroni-corrected post-hoc analyses did not reveal significant differences in MMN amplitude strength with the exception of the comparison of CM vs. NM for the MMN amplitude to slide (p = 0.020), CM having stronger MMN than NM.

The overall ANOVA revealed significant within-subjects effect of Feature (*F*_5, 445_ = 37.0, *p* < 0.0001, *η*_*p*_^*2*^ = 0.294) with the strongest MMN elicited to the location deviant and the lowest to the intensity and rhythm deviants. Stronger MMN amplitudes were recorded from the right electrodes than from the left ones (Laterality: *F*_1, 89_ = 9.22, *p* = 0.003, *η*_*p*_^*2*^ = 0.094) and from the frontal electrodes as compared to the central electrodes (Frontality: *F*_1, 89_ = 40.9, *p* = < 0.0001, *η*_*p*_^*2*^ = 0.315). There was also a significant interaction Feature × Laterality (*F*_5, 445_ = 6.65, *p* = < 0.0001, *η*_*p*_^*2*^ = 0.070) suggesting that MMN amplitude to pitch, timber and rhythm deviants was significantly stronger in the right hemisphere than in the left (p < 0.05).

### MEG data

We observed a significant main effect of Group on MMNm amplitude (*F*_3, 98_ = 6.33, *p =* 0.001, *η*_*p*_^*2*^ = 0.162), resulting from an overall larger MMNm in JM as compared to that in NM (p < 0.0001) and AM (*p* = 0.032).

**Fig 3:**
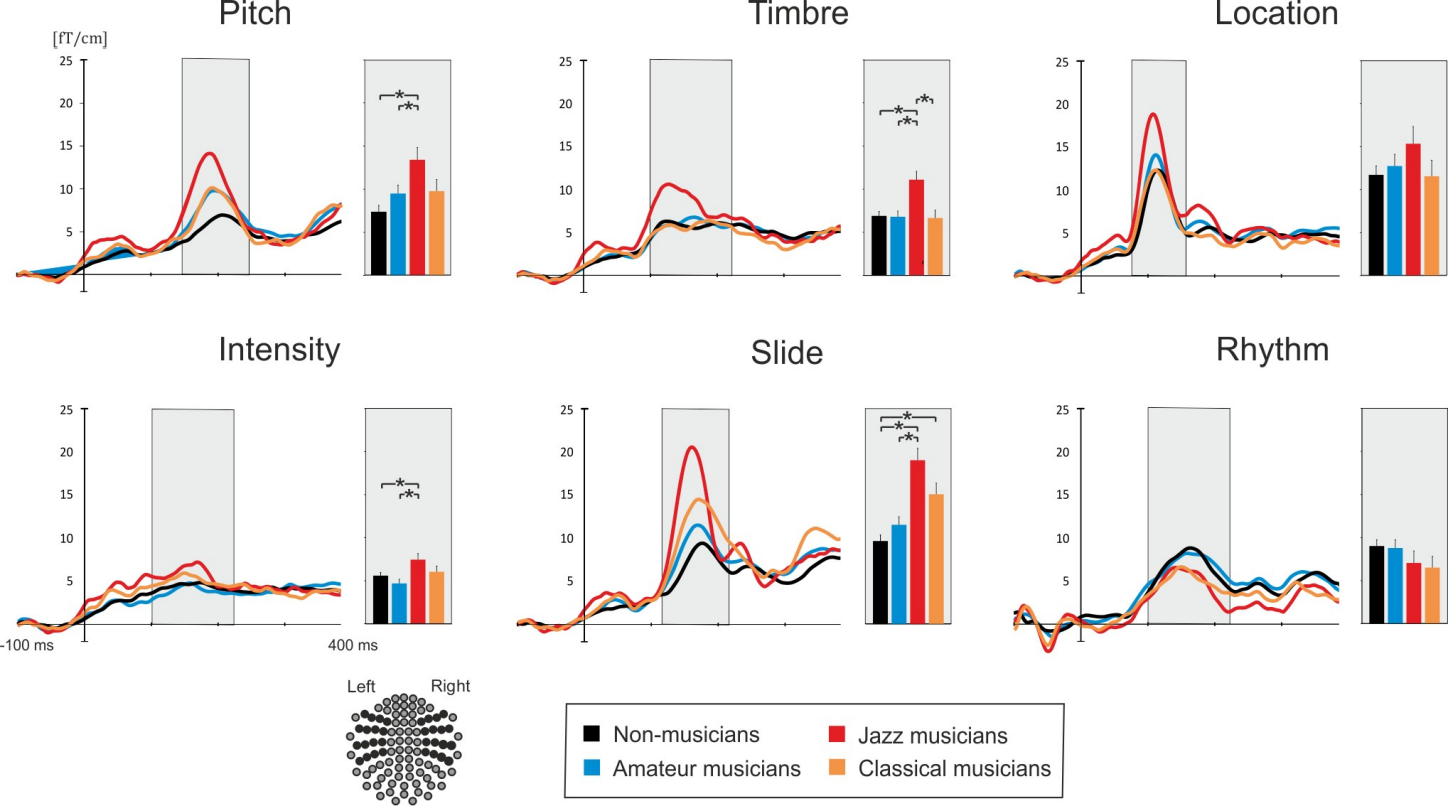
Average areal mean curves and MMNm amplitudes obtained for each musical group and six types of deviants. Histograms represent mean values of MMNm responses. Asterisks mark significant results of Bonferroni-corrected planned comparisons (*p* < 0.05). A schematic picture of the gradiometers selected for calculating areal mean curves (dark circles) is at the bottom left.

The comparison of MMNm amplitudes to different deviant types revealed a significant effect of Feature (*F*_5, 490_ = 44.77, *p* < 0.0001, *η*_*p*_^*2*^ = 0.314) with the strongest MMNm amplitudes obtained for the location and slide deviants, while the smallest responses were registered to the intensity deviant. Furthermore, there was a Feature x Group interaction (*F*_15, 5010_ = 4.07, *p* < 0.0001, *η*_*p*_^*2*^ = 0.107) that suggested group differences in MMNm amplitude to different sound deviations. In separate follow-up ANOVAs, we found that the observed interaction was driven by group differences in pitch, timbre, intensity and slide MMNm (Fig 3; for statistics see Table 4). For each of these deviants, JM had a stronger MMNm than NM and AM (*p*s ≤ 0.042), whereas CM had a significantly larger amplitude of MMNm only as compared to that of NM for the slide deviant (p = 0.001). Moreover, timbre MMNm was significantly stronger in JM than in CM (p = 0.002).

**Table 4:** ANOVA results for the MMNm amplitudes.

Feature	*dF*	*F*	*p*	*η*_*p*_^*2*^
Pitch	3, 99	4.78	**0.004**	0.127
Timbre	3, 99	6.49	**0.0004**	0.164
Location	3, 99	1.69	0.175	0.049
Intensity	3, 99	3.74	**0.014**	0.102
Slide	3, 99	15.09	**< 0.0001**	0.316
Rhythm	3, 99	1.34	0.264	0.039

Significant *p-*values are shown in bold.

In general, MMNm amplitude recorded in the right hemisphere was larger than in the left (Laterality: *F*_1,98_ = 52.60, *p* < 0.0001, *η*_*p*_^*2*^ = 0.349). MMNm distribution was also dependent on the type of the deviation as evident from the significant interactions Feature × Laterality (*F*_5, 490_ = 3.90, *p* = 0.007, *η*_*p*_^*2*^ = 0.038).

Musicians are known to develop sensitivity to the sound of their own instrument [36] and since we used stimuli played with piano sound, we performed an additional analysis contrasting pianists with other instrumentalists irrespective of their genre identity to find out if expertise in piano could be a potential confound in our results. We performed ANOVAs contrasting (1) musicians playing piano as their main instrument vs all other musicians (N = 9 and 19, respectively), and (2) musicians playing piano as their main or secondary instrument vs all other musicians (N = 17 and 11, respectively). In both analyses, factor Group was not found significant (p = 0.843 in the first contrast, and p = 0.524 in the second contrast) and did not interact with either Feature or Laterality factors in within-subject comparisons (*p* > 0.05 for all). Nevertheless, the significant main effect of Group is obtained when JM and CM as used in the original analysis are contrasted (*F*_1, 26_ = 9.13, *p* = 0.006, *η*_*p*_^*2*^ = 0.260) with JM showing stronger MMNm than that of CM.

### Correlations between MMN and style preferences

**Fig 4:**
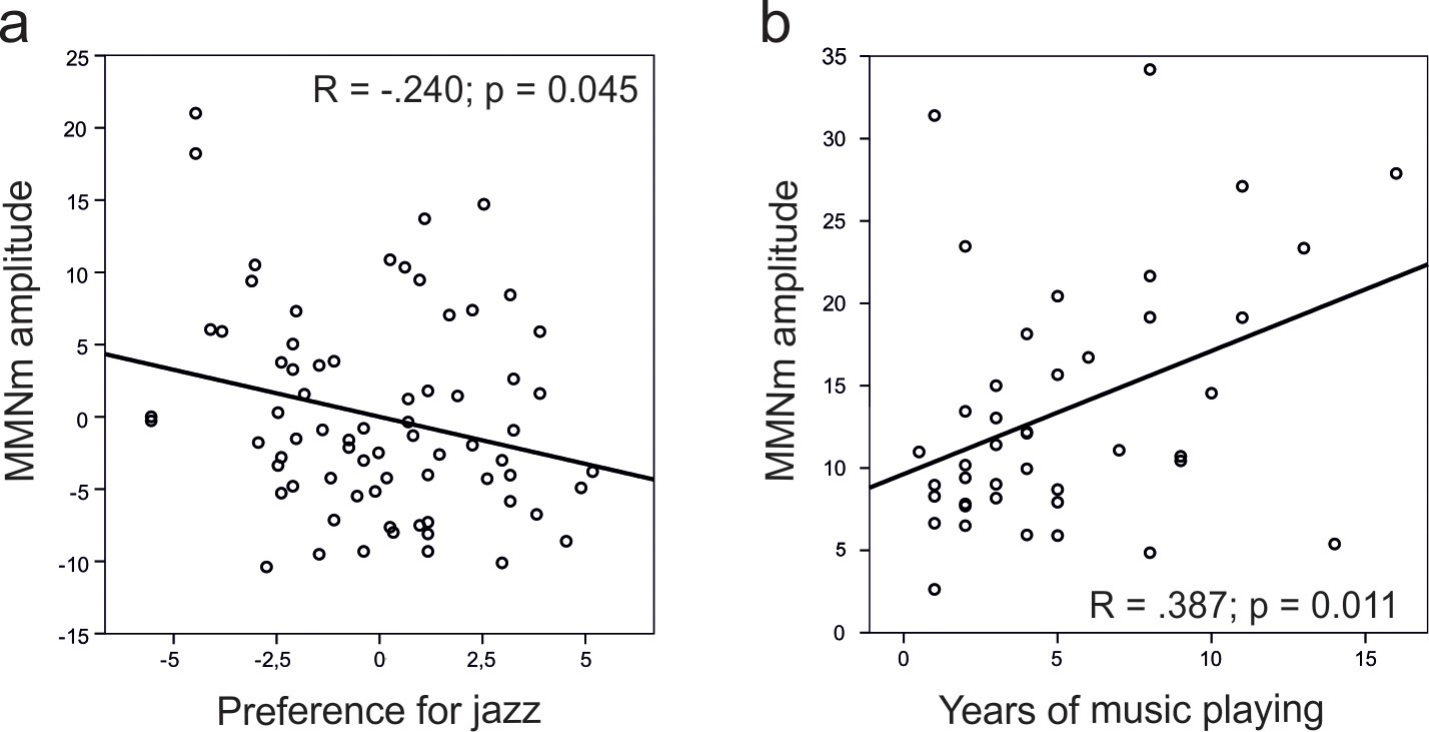
Correlation plots. (a) Significant partial correlation between right hemisphere MMNm amplitudes to slide and preference for jazz music (left) in non-musicians and amateurs (NM + AM) controlled for preference for classical music (corrected data plotted); (b) Significant correlation between years of music playing and right hemisphere MMNm amplitude to slide.

Having shown that practicing jazz or classical music has a differential effect on the ability to discriminate sound feature changes, we asked if a mere preference for these musical styles has any effect on discrimination ability in NM and AM. For that, we concentrated on MMNm responses for which we found Group effect, namely pitch, slide, timbre, and intensity. As the most prominent, only the right hemisphere MMNm was used for this analysis. We found that in the joint group of NM and AM, the slide MMNm amplitude correlated with preference for jazz music (r_part_ = -0.240, p = 0.045; Fig 4A) while controlling for the classical music preference and not vice versa. The direction of the correlation was such that the more subjects preferred jazz music, the smaller MMNm to slide they exhibited. None of the other partial correlations showed a significant relationship between a style preference and an MMNm amplitude. The correlations between MMN to slide and preference for jazz music are illustrated in S1 Fig.

To delineate whether the experience with playing music that some of the participants had (measured in years) could contribute to the observed negative correlation between preference for jazz music and MMNm to slide, we performed a correlation analysis. While years of playing music did not correlate with preferences for jazz or classical music (p < 0.05), they were positively correlated with the amplitude of MMNm to the slide deviant (r = .387, p = 0.011) among participants with at least one year of musical training (N = 42; Fig 4B). Thus, an opposite direction to the correlation with the preference for jazz music was observed.

## Discussion

In accordance with our hypotheses, we found that active listening experience in professional musicians enhances neuronal prediction errors above and beyond the effect of just listening to music. Specifically, in addition to an overall increased MMN amplitudes to the deviants, jazz musicians displayed greater MMN to slide than other groups. Indeed, this result was confirmed even with only few years of musical experience since we also found a positive correlation between years of training and slide MMN amplitudes in amateur musicians and non-musicians. However, in these participants their slide MMN was negatively correlated with the preference for jazz but not classical music. Pairing these results with the fact that groups did not vary from each other in weekly time spent listening to music, we propose that active engagement with and formal knowledge of a musical style are crucial for developing accurate priors that inform auditory-cortex discrimination of the sound features of the preferred style, in contrast to just listening experience with a preferred musical repertoire.

Attaining musical expertise requires developing a number of perceptual and motor skills, and a strong motivation for maintaining intensive and frequent practice [53]. Furthermore, engagement with a certain musical style, thus actively training its typical features and prerequisites, differentiates musicians in different styles of music from each other [22,37,38]. Consistent with this, we found differences in neural discrimination of sound feature changes, particularly in pitch, pitch slide, timbre, and intensity in professional musicians depending on whether they practiced and performed jazz or classical music. Both groups showed heightened discrimination of pitch slide as compared to non-musicians, and the jazz group’s MMN exceeded amateur musicians’ as well. However, only jazz musicians, but not classical musicians, had enhanced neural discrimination of pitch and intensity as compared to non-musicians and amateurs. The MMN to timbre change was strongly enhanced in jazz musicians compared to all other groups. Greater sensitivity of jazz musicians to changes in musical feature is in accordance with findings of Vuust and colleagues [37], and advocates for generally higher skills of jazz musicians in discriminating rapid changes in music. This may relate to the nature of jazz and its tradition, where improvisation, and thus the ability to quickly evaluate and respond in an interactive manner to the music produced by others, plays a major role and places a demand on a specific training [54]. Seppänen et al. [55] showed that musicians who improvised and practiced playing by ear had enhanced neural discrimination of intervals and melody contour changes (as indexed by the MMN error signal) as compared to musicians who used scores in their musical practice.

We attribute the observed group differences in professional musicians to the influence of long-term practice and knowledge of particular musical styles as they had comparable years of musical experience and weekly time spent playing music. Allowance should be made, though, for the fact that certain instruments are more common in different musical styles over others, such as piano in classical music or saxophone in jazz music. Since auditory feature processing is sensitive to the timbre of the instrument practiced [33,36], a prevalence of musicians playing the same instrument in one group over another could influence the results. However, in the current study, the majority of the participants were multi-instrumentalists and there was no bias towards one type of musical instrument musicians played and experience with playing piano either as the main or secondary instrument did not gave advantages in feature discrimination to musicians in our study. Hence, we infer that the instrument played did not drive the differences in sound feature discrimination skills within musicians, and we rather can attribute the observed group differences to the practiced musical styles.

However, our findings on the associations between MMNm amplitudes for slide deviants and preferences for jazz music warrant a special place for discussion. Through familiarization with a preferred musical style, an individual also familiarizes with its instruments and acoustic and stylistic features, thus building a wider ‘vocabulary’ of different sound features. Thus, listening to music of a particular style reduces the ‘novelty’ of features comprising it. Sliding pitch is a common feature of improvisational music, so individuals preferring jazz music could be well accustomed to sliding sounds and accommodate them in their model of music sound expectations. In this case, MMN amplitude, thought to reflect an error signal of expectation violation, decreasing with a higher preference for jazz music could indicate the better familiarization that jazz enthusiasts have with sliding pitch, as one of the common attributes of their musical environment. Conversely, actively trained musicians are much more familiar with specific instruments and their physical constraints, potentially making them more sensitive to the sliding piano tones in this paradigm since piano sounds do not normally slide. In other words, while jazz involves many sliding tones played by several instruments (but not by piano), more-trained musicians might better understand that these slides typically do not come from pianos, and so they show less tolerance to manipulations with its sound. Thus, the MMN to the slide deviant in musicians is likely facilitated because of them having formal knowledge of musical rules in addition to having intensive motor practice and being accustomed to a wide range of sound features.

Based on neurophysiological studies with human subjects, Kraus and Chandrasekaran [56] suggest that musical practice leads to selective adaptations to the fine-grained perception of important features of auditory information developed at a different extent according to the relative importance of these features for musical practice. Professional musicians learn to link musical sounds to their meaning starting from an early age, so they gain a unique listening experience with the music they intensively practice and perform. In the absence of formal training and regular musical practice, the sound features of a preferred musical style might not hold a specific meaning for a non-professional listener [56]. The importance of sensory-motor involvement in music was shown in a study with two weeks of practicing short melodies on the piano, leading to greater improvements in melody change discrimination compared to just listening to the same melodies [57]. Similarly, learning a non-native language in adulthood with little emphasis on pronunciation does not improve the neuronal discrimination of vowels typical for the second language [58]. This could explain why the non-musicians and amateurs with less active musical training have smaller MMN amplitudes for slides, even as smaller MMN amplitudes to slides correlate with stronger jazz preferences: listeners with more training in general have more sensory-motor associations that facilitate neuronal discrimination, while those with more specific jazz-listening experience are more accustomed to slides and thus less sensitive to them.

### Limitations

One limitation of the current study is an uneven distribution of male and female subjects in jazz and classical musician groups. Future studies should attempt recruiting a gender-matched sample of subjects despite that this imbalance seems to be the actual representation of genders in these musical genres [37,38] and thus a balanced sample may remove inherent differences between musicians of different types and undermine the effects of variable musical profiles put under investigation [38].

However, the difference in gender distribution could have an influence on MEG findings in our study due to the neuromagnetic signal being sensitive to sensor-to-head center distance. MEG helmets use a fixed sensor array optimized to fit most of the adult heads, however, there is a natural difference in the average head size of males and females and thus in the distance between the cortical sources and MEG sensors. It could be so that higher amplitude of MMNm responses in jazz musicians with the majority of male subjects could be related to a lesser decay of the MEG signal power due to larger average head size in this group.

This fact could also be a potential contribution to the difference in the results obtained from EEG and MEG modalities, where the group differences were more pronounced in the latter. However, since MMNm findings of the current study were in line with previous findings of enhanced automatic discrimination skills in jazz musicians obtained with EEG [37], we attribute the lack of significant group differences in MMN after the correction for multiple comparisons to a lower signal-to-noise ratio in EEG vs MEG [44] as well as higher frequency rate of deviant presentation in the no-standard as compared to other versions of the musical multifeature MMN paradigm used in previous studies [37,41,59,60].

It is also important to note that a novel finding of the present study, that is in the negative relationship between preference for jazz music and the amplitude of MMNm to a sliding pitch, was obtained in a combined group of non-musicians and amateur musicians where the genders were evenly distributed. Importantly, the relationship between MMN(m) and preference for jazz music was present in both MEG and EEG modalities (see S1 Fig). Since the EEG signal is not affected by head sizes, we argue that the central finding of this study did not result from the differences in signal strength.

### Conclusions

We conclude that priors learned from active vs. passive engagement with a musical style shape the auditory-cortex responses to deviations of spectral (rather than temporal) features inserted in an ever-changing fast musical sequence. These effects are closely dependent on how priors are derived from past listening experience, whether active such as in professional musicians or passive such as in casual music listeners. Our findings showed differential effects of passive preference to a musical style and active practice as well as explicit knowledge of the relevant style on neural responses to a spectral feature deviation. As such, professional jazz musicians developed more accurate discrimination of pitch, pitch slide, timbre and intensity changes. On the other hand, a higher preference for a musical style in individuals with no or little musical training was associated with reduced neuronal response to pitch slide, which is the opposite to the effect of music playing experience in the same population. This suggests that active experience of a musical style is crucial for developing accurate priors and consequently an enhanced automatic neural discrimination of the sound features of the preferred style, in contrast to a passive experience of it.

## Supporting information

S1 Table Group performance on musicality tests and correlation with MMN. (DOCX)

S1 Fig Correlation plots. Correlations between MMN amplitudes to slide and preference for jazz music in non-musicians and amateurs (NM + AM).
(TIF)
