# Supplementary material for: Fractionating auditory priors: A neural dissociation between active and passive experience of musical sounds
Source: PLoS One. 2019 May 3;14(5):e0216499. doi: 10.1371/journal.pone.0216499 (PMC6499420; doi:10.1371/journal.pone.0216499)
Supplement: S1 Table — (DOCX) [file pone.0216499.s001.docx]

| **S1 Table**  **Group performance on musicality tests and correlation with MMN** | | | | | | | | | | | | |
| --- | --- | --- | --- | --- | --- | --- | --- | --- | --- | --- | --- | --- |
| **Test** | **ANOVA** | | |  | | **Descriptives** | | | | | **Correlation with MMN*** | |
|  | ***df*** | ***F*** | ***p*** | **Group** | **N** | | **Mean** | **SD** | **Min** | **Max** | **r** | ***p*** |
| On-line Test: Scale | 3 | 7.89 | **0.0002** | NM | 27 | | 82.9 | 10.9 | 57 | 100 | 0.286 | 0.112 |
|  |  |  |  | AM | 16 | | 90.9 | 5.4 | 80 | 100 |  |  |
|  |  |  |  | JM | 7 | | 94.4 | 5.3 | 83 | 97 |  |  |
|  |  |  |  | CM | 10 | | 95.1 | 4.3 | 87 | 100 | -0.254 | 0.161 |
| On-line Test: Beat | 3 | 6.55 | **0.001** | NM | 27 | | 81.9 | 7.2 | 71 | 96 |  |  |
|  |  |  |  | AM | 16 | | 85.6 | 8.2 | 71 | 96 |  |  |
|  |  |  |  | JM | 7 | | 90.1 | 7.9 | 75 | 100 |  |  |
|  |  |  |  | CM | 10 | | 93.1 | 6.2 | 83 | 100 |  |  |
| On-line Test: Out of Key | 3 | 2.07 | 0.115 | NM | 27 | | 82.6 | 10.0 | 54 | 96 | 0.125 | 0.497 |
|  |  |  |  | AM | 16 | | 90.8 | 9.8 | 71 | 100 |  |  |
|  |  |  |  | JM | 7 | | 90.7 | 6.3 | 79 | 96 |  |  |
|  |  |  |  | CM | 10 | | 86.8 | 18.6 | 50 | 100 |  |  |
| Seashore Pitch | 3 | 1.72 | 0.176 | NM | 19 | | 41.7 | 6.4 | 28 | 50 | 0.282 | 0.117 |
|  |  |  |  | AM | 16 | | 43.1 | 5.5 | 27 | 50 |  |  |
|  |  |  |  | JM | 8 | | 45.8 | 5.1 | 34 | 50 |  |  |
|  |  |  |  | CM | 8 | | 46.5 | 5.2 | 35 | 50 |  |  |
| Seashore Time | 3 | 1.25 | 0.303 | NM | 19 | | 42.7 | 2.8 | 37 | 48 | -0.154 | 0.400 |
|  |  |  |  | AM | 16 | | 43.6 | 4.0 | 35 | 48 |  |  |
|  |  |  |  | JM | 8 | | 44.4 | 3.7 | 39 | 48 |  |  |
|  |  |  |  | CM | 8 | | 45.3 | 2.5 | 41 | 48 |  |  |
| *Correlation between the amplitude of the right MMNm to slide and a test score was tested in a joint group of non-musicians and amateur musicians.  Significant *p-*values are shown in bold. NM = non-musicians; AM = amateur musicians; JM = jazz musicians; CM = classical musicians. | | | | | | | | | | | | |
