# Supplementary figures and images for: Fractionating auditory priors: A neural dissociation between active and passive experience of musical sounds
Source: PLoS One. 2019 May 3;14(5):e0216499. doi: 10.1371/journal.pone.0216499 (PMC6499420; doi:10.1371/journal.pone.0216499)

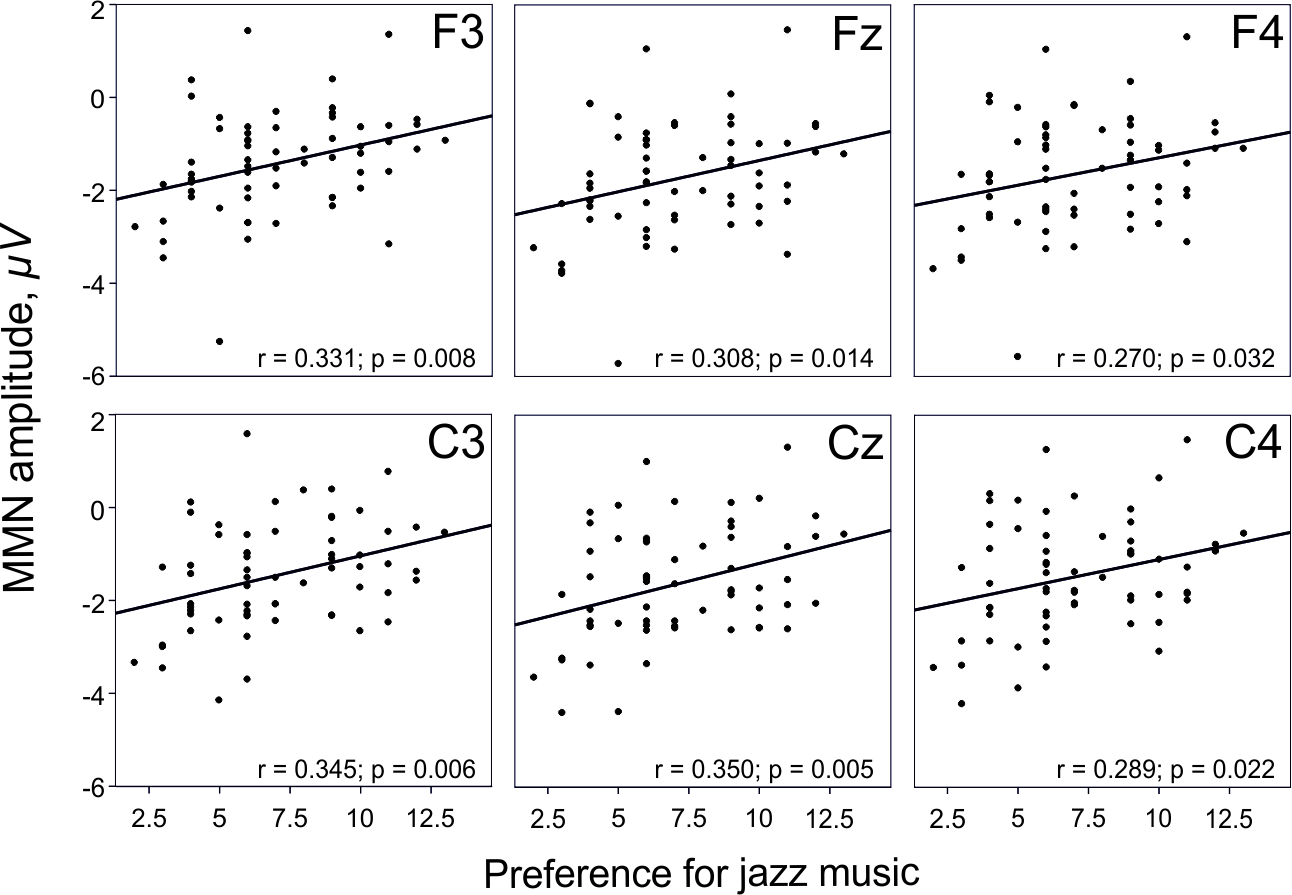

Supplement: S1 Fig — Correlations between MMN amplitudes to slide and preference for jazz music in non-musicians and amateurs (NM + AM). (TIF) [file pone.0216499.s002.tif]
